# Natural Oncolytic Activity of Live-Attenuated Measles Virus against Human Lung and Colorectal Adenocarcinomas

**DOI:** 10.1155/2013/387362

**Published:** 2013-03-17

**Authors:** Nicolas Boisgerault, Jean-Baptiste Guillerme, Daniel Pouliquen, Mariana Mesel-Lemoine, Carole Achard, Chantal Combredet, Jean-François Fonteneau, Frédéric Tangy, Marc Grégoire

**Affiliations:** ^1^INSERM, UMR892, 44000 Nantes, France; ^2^CNRS, UMR6299, 44000 Nantes, France; ^3^Université de Nantes, 44000 Nantes, France; ^4^Unité de Génomique Virale et Vaccination, CNRS-URA 3015, Institut Pasteur, 75015 Paris, France

## Abstract

Lung and colorectal cancers are responsible for approximately 2 million deaths each year worldwide. Despite continual improvements, clinical management of these diseases remains challenging and development of novel therapies with increased efficacy is critical to address these major public health issues. Oncolytic viruses have shown promising results against cancers that are resistant to conventional anticancer therapies. Vaccine strains of measles virus (MV) exhibit such natural antitumor properties by preferentially targeting cancer cells. We tested the ability of live-attenuated Schwarz strain of MV to specifically infect tumor cells derived from human lung and colorectal adenocarcinomas and demonstrated that live-attenuated MV exhibits oncolytic properties against these two aggressive neoplasms. We also showed that Schwarz MV was able to prevent uncontrollable growth of large, established lung and colorectal adenocarcinoma xenografts in nude mice. Moreover, MV oncolysis is associated with *in vivo* activation of caspase-3 in colorectal cancer model, as shown by immunohistochemical staining. Our results provide new arguments for the use of MV as an antitumor therapy against aggressive human malignancies.

## 1. Introduction

Lung and colorectal cancers are leading causes of death worldwide with approximately 1.6 million and 1.2 million new cases per year resulting in 1.4 million and 610,000 estimated deaths, respectively [1]. In developed countries, lung cancers rank first for males and second for females in overall cancer-related deaths while colorectal cancers rank second for male and third for female. These cancers are found to be extremely resistant to conventional therapies including surgery, chemotherapy, and radiotherapy with a 5-year survival of only 15% and 50%, respectively. 

Difficult clinical management of these two malignancies makes them ideal candidates for development of alternative approaches such as cancer virotherapy [2–5]. Live-attenuated vaccinal strains of measles virus (MV) are of particular interest due to their ability to specifically target different types of human tumors [6, 7] through recognition of the CD46 membrane complement regulatory molecule [8, 9] which is frequently overexpressed on cancer cells [10]. Oncolytic viruses are also powerful inducers of tumor cell death and thus could help to cure cancers that are refractory to conventional treatments. Furthermore, in addition to inducing cell death, the infection of tumor cells by MV is able to activate components of the antitumor immune response, such as myeloid and plasmacytoid dendritic cells, that may play a role in the efficacy of cancer virotherapy [6, 11].

Several Phase I clinical trials targeting different cancers with MV virotherapy are in progress and two of them have been published. In the initial Phase I clinical trial, intratumoral injection of low doses of MV into five patients with cutaneous-T-cell-lymphoma, patients induced stabilization of the disease in two patients and a partial response in one [12]. The authors of the second published Phase I clinical trial, carried out in patients with refractory ovarian cancers [13], noticed a dose-dependent biological activity of oncolytic MV. They also reported that the treatment was well tolerated, thus confirming previous reports that demonstrated the safety of using live-attenuated vaccinal strains of MV in the clinical setting.

Sparse data from the literature show that wild-type MV is able to infect some human lung and colorectal adenocarcinoma cells [14, 15]. MV fusogenic membrane glycoproteins by themselves were also demonstrated to improve treatment of human colorectal cancer xenografts when used in combination with chemotherapy or virotherapy [16]. Moreover, urokinase receptor-retargeted MV was shown to display oncolytic activity against murine colorectal cancer cells *in vivo* [17]. However, whereas MV virotherapy has been tested against a wide variety of human cancers, no comprehensive work has been achieved until now to investigate how oncolytic strains of MV target human lung and colorectal adenocarcinomas.

To our knowledge, we demonstrate here for the first time that live-attenuated Schwarz vaccinal strain of MV is able to specifically infect and kill tumor cells derived from human lung and colorectal adenocarcinomas, both *in vitro* and *in vivo* against large tumor burdens. Specifically, these oncolytic properties are associated with *in vivo* activation of caspase-3. Altogether, our results confirm the ability of oncolytic MV to target aggressive neoplasms and thus provide new perspectives for the treatment of two major malignancies.

## 2. Materials and Methods

### 2.1. Cell Culture

ADK3, ADK117, and ADK153 lung adenocarcinoma cell lines were established in our laboratory from pleural effusions collected by thoracocentesis of cancer patients, with their informed consent, and genetically characterized [18]. The A549 lung adenocarcinoma, Caco-2, HT29, SW480, and SW620 colorectal adenocarcinoma cell lines were purchased from ATCC. All cell lines were maintained in RPMI-1640 medium (Gibco-Invitrogen, Cergy-Pontoise, France) supplemented with 10% (v/v) heat-inactivated fetal calf serum (PAA Laboratories, Les Mureaux, France), 2 mM L-glutamine, 100 U/mL penicillin, and 100 *μ*g/mL streptomycin (all purchased from Gibco). Normal bronchial epithelial cells (BEC) were obtained from a healthy lung graft (Dr Magnan, INSERM UMR 915, Nantes) and cultured in CnT-17 medium (CELLnTEC, Switzerland). Cells were cultured at 37°C in a humidified, 5% CO_2_ atmosphere and were routinely checked for *Mycoplasma* contamination by PCR.

### 2.2. *In Vitro* Measles Virus Infection

Live-attenuated Schwarz vaccinal strain of measles virus recombinant for enhanced green fluorescent protein (MV-eGFP) was produced as previously described [19] and titered on Vero cells (TCID_50_/mL). *In vitro* infections were performed at MOI = 1.0 TCID_50_ for 2 h at 37°C. Viral inoculum was then replaced by fresh culture medium with no further renewal during the experiments.

### 2.3. Time-Lapse Microscopy

All experiments were performed at the Cellular and Tissular Imaging Core Facility (MicroPICell, IFR26, Nantes, France) using a Leica DMI6000B station. Images were acquired every 15 min for 72 hours with MetaMorph Microscopy Automation & Image Analysis Software (Molecular Devices) and further treated with ImageJ (National Institute of Health).

### 2.4. Cell Death Analysis

Percentages of dying cells were determined 3 days after infection using the Apoptosis Detection Kit (BD Biosciences) following manufacturer's instructions. Briefly, cells were double stained with annexin-V-FITC and propidium iodide (PI) for 15 min and analyzed by flow cytometry within 1 hour.

### 2.5. Flow Cytometry

Cells were incubated for 30 min with FITC-conjugated anti-CD46, PE-conjugated anti-CD150/SLAM (BD Biosciences, Le Pont de Claix, France), or PE-conjugated anti-Nectin-4 (R&D Systems Europe, Lille, France) antibodies in PBS/0.1% BSA for extracellular staining. Cells were then washed 3 times with PBS before analysis by flow cytometry (FACSCalibur, BD Biosciences).

### 2.6. Animal Model and *In Vivo* Experiments

All *in vivo* experiments complied with European Union regulations on the welfare and use of animals in cancer research. Six-week-old female *RJ:NMRi-nu *nude mice were purchased from Centre d'Élevage Janvier (Le Genest-Saint-Isle, France). Mice were challenged subcutaneously with 10^6^ tumor cells in the left flank. When volumes reached approximately 150–200 mm^3^ for Caco-2 or 100 mm^3^ for A549, MV-eGFP (1.5 × 10^7^ TCID_50_) or saline buffer (PBS) was injected intratumorally (50 *μ*L). Tumors were measured twice weekly with a microcaliper and tumor volumes were calculated using the (length^2^ × breadth)/2 formula. Animals were sacrificed when tumors reached 1-2 cm^3^ in volume. Tumors were then harvested, fixed in 4% paraformaldehyde (Electron Microscopy Sciences, Hatfield, USA) and embedded in paraffin wax.

### 2.7. Immunohistochemistry

Immunohistochemical stainings were performed by the MicroPICell core facility with a Bond Max automaton (Menarini, Rungis, France). Briefly, paraffin-embedded tumor slides were incubated in a demasking citrate buffer (pH = 6.0) before blocking of endogenous peroxidase for 5 min. Slides were then incubated for 1 h with a polyclonal, rabbit antiactive-caspase-3 antibody (Abcam, Paris, France) diluted to 1 : 50 and subsequently with Histofine Rabbit-to-Mouse secondary antibody (Nichirei Biosciences, Tokyo, Japan). After 10 min 3,3′-Diaminobenzidine (DAB) incubation, tumors were counterstained with hematoxylin. Slides were analyzed using a Leica DM2500 microscope coupled to a Leica DCF295 camera. Images were acquired with Leica Application Suite Version 3 software.

### 2.8. Statistical Analyses

One-sided, unpaired Mann-Whitney *t*-test was used to compare groups in the *in vivo* experiments. Differences were considered significant when *P < 0.05 or **P < 0.01. All data are presented as mean ± SEM.

## 3. Results

### 3.1. Human Adenocarcinoma Cells Are Efficiently Infected by MV

**Figure 1:**
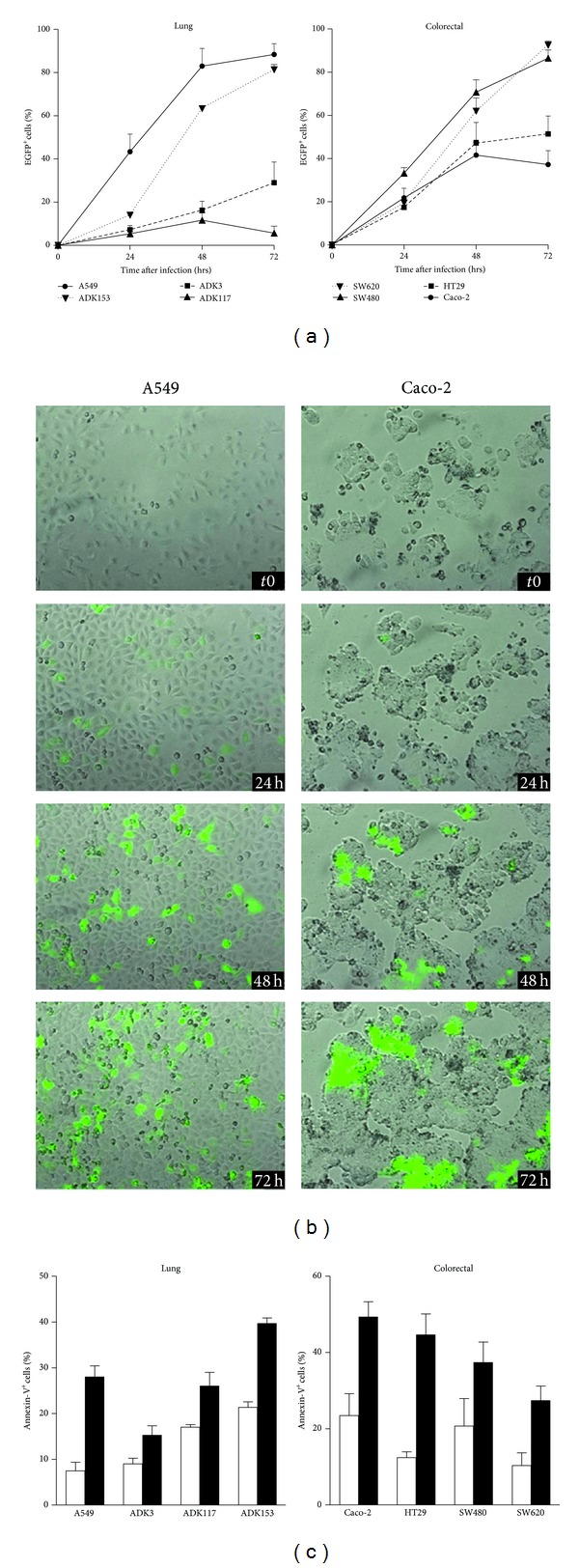
*In vitro* oncolytic properties of measles virus against human adenocarcinomas. (a) One million human lung or colorectal adenocarcinoma cells were infected *in vitro* with a live-attenuated strain of MV recombinant for EGFP (MV-eGFP; MOI = 1). Percentages of infected cells (EGFP^+^) were determined by flow cytometry at 24, 48, and 72 h after infection. Data are presented as mean ± SEM, (*n* = 3). (b) Infected cells were observed for 72 h by time-lapse microscopy to study cytopathic effects of MV infection. Complete experiments are presented in Figures S1 (Caco-2) and S2 (A549) in Supplementary Material available online at http://dx.doi.org/10.1155/2013/387362. (c) Human lung and colorectal adenocarcinoma cells were either infected with live-attenuated MV (MOI = 1, black bars) as described above or left uninfected (white bars). Cells were cultured for 72 h without medium renewal. Cells were then double stained with annexin V/propidium Iodide and analyzed by flow cytometry.

Cancer cells derived from human lung and colorectal adenocarcinomas were first infected with live-attenuated Schwarz strain of MV recombinant for EGFP (MV-eGFP) to test their sensitivity to oncolytic MV. We infected four different colorectal adenocarcinoma cell lines and showed that they were all susceptible to MV infection (Figure 1(a)). Whereas SW480 and metastatic SW620 cells showed high infection yield after 3 days with 87% and 93% of cells infected, respectively, we observed less EGFP^+^ HT29 (51%) and Caco-2 (37%) cells at the same time point. To determine how MV infection spreads in these two cell lines, we further analyzed MV infection by time-lapse microscopy. In Caco-2 culture, infection progressed slowly despite tight cell interactions and typical syncytia formation resulting from fusion of infected tumor cells with neighboring cells (Figures 1(b) and S1). On the other hand, HT29 cells underwent a rapid oncolytic process with infected cells dying shortly after infection (not shown). We observed with fluorescent microscopy that HT29 cell death resulted in the release of EGFP in the extracellular medium, especially at 72 h, thus lowering the amount of EGFP^+^ cells determined by flow cytometry (not shown).

We also tested oncolytic properties of MV against one commercial (A549) and three (ADK3, ADK117, and ADK153) lung adenocarcinoma cell lines obtained in our laboratory. These cell lines exhibited heterogeneous infection rates following exposure to MV-eGFP. While A549 and ADK153 cells were efficiently infected with 88% and 65% EGFP^+^ cells after 72 h, respectively, infection spread slowly in ADK3 cells with only 29% of infected cells at the same time (Figure 1(a)). ADK117 cells were found to be resistant to infection with only 6% of infected cells at 72 h after infection. Time-lapse experiments confirmed efficient infection of A549 cells by MV, even though only minimal syncytia formation was observed (Figures 1(b) and S2). On the contrary, normal bronchial epithelial cells (BEC) were not infected by MV (0.4% at 72 h, MOI = 1, data not shown). Thus, our results show that 4 out of 4 colorectal and 3 out of 4 lung adenocarcinoma tested cell lines, but not normal epithelial cells, are sensitive to live-attenuated MV infection.

### 3.2. MV Induces Cell Death of Infected Lung and Colorectal Cancer Cells

To further investigate oncolytic properties of MV against lung and colorectal cancer cells, we studied the ability of the virus to induce death of the infected cells. We initially characterized MV-related cytopathic effects by fluorescence microscopy. As expected, MV infection induced the formation of giant, multinucleated cells, namely, syncytia, in colorectal adenocarcinoma cells (Figures 1(b) and S1). Ultimately, MV infection caused detachment of MV-induced syncytia from the plate support, thereby demonstrating the induction of tumor cell death by MV infection. Infected A549 lung adenocarcinoma cells only formed small syncytia resulting from fusion of two to five tumor cells, though these cells were eventually driven to apoptotic-like cell death, as shown by consistent observations of plasma membrane blebbing (Figures 1(b) and S2).

To better characterize the cell death induced after MV infection, we performed annexin-V/propidium iodide double staining (Figure 1(c)). Oncolytic MV infection efficiently induced cell death in A549, ADK153, Caco-2, HT29, SW480, and metastatic SW620 cells as shown by a substantial increase of annexin-V^+^ cell percentages compared to uninfected cells. Conversely, death induction in ADK3 and ADK117 cells was minimal, consistent with the relatively low infection rates observed in previous experiments (Figure 1(a)). Thus, analysis of cell death induction demonstrates that oncolytic MV effectively kills infected cancer cells derived from human lung and colorectal adenocarcinomas through an apoptotic-like process.

### 3.3. CD46 Is Highly Expressed in Lung and Colorectal Cancer Cell Lines

**Figure 2:**
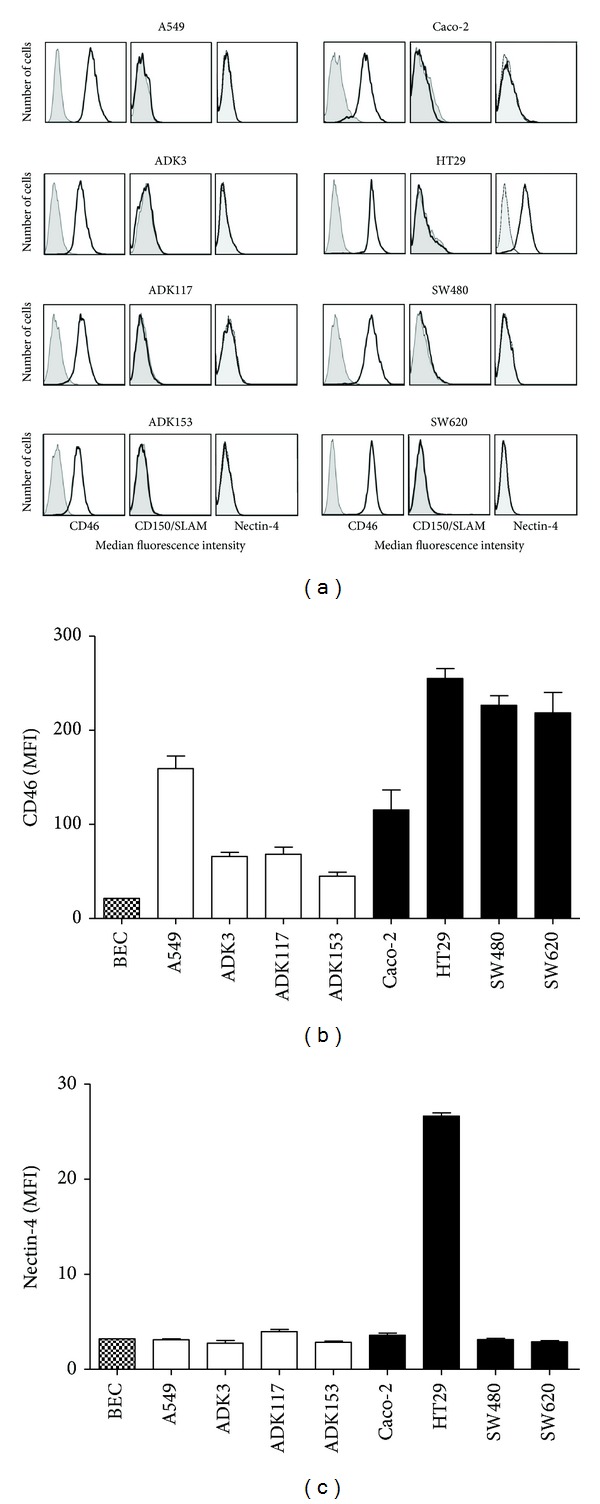
Expression of CD46 and CD150/SLAM receptors on human adenocarcinoma cells. (a) Lung (left) and colorectal (right) tumor cell lines were stained with anti-CD46-FITC, anti-CD150/SLAM-PE, and anti-Nectin-4-PE antibodies (thick black lines) in PBS/0.1% BSA for 30 min before analysis by flow cytometry. Isotypic stainings are shown as grey filled curves. (b)-(c) CD46 (b) and Nectin-4 (c) expression levels were determined for each lung (white bars) or colorectal (black bars) tumor cell line in three independent experiments. Expression levels of CD46 and Nectin-4 for normal bronchial epithelial cells (BEC) are indicated. MFI: median fluorescence intensity.

Despite recent advances in the identification of MV cellular receptors [14, 20], CD46 is still considered to be critical in determining the sensitivity of tumor cells to oncolytic strains of MV. As expected, a majority of efficiently infected tumor cells exhibited a high CD46 surface level (Figures 2(a) and 2(b)). Indeed, A549 (MFI = 159), Caco-2 (115), HT29 (255), SW480 (227), and SW620 (219) were efficiently infected and killed, which is consistent with their high expression of the CD46 receptor. Interestingly, CD46 expression analysis was not sufficient to predict the infection levels of some lung adenocarcinoma cell lines. For instance, fairly high levels of CD46 were expressed on ADK3 (MFI = 66) and ADK117 (68) but these cells were less efficiently infected than ADK153 cells which exhibit low CD46 expression (MFI = 45). Consistent with their resistance to infection, normal bronchial epithelial cells barely expressed CD46 (MFI = 21).

To better determine which receptor(s) were involved in the infection of these lung adenocarcinoma cell lines, we subsequently screened them for expression of CD150/SLAM (Signaling Lymphocytic Activation Molecule) and Nectin-4 receptors. CD150/SLAM is known to be involved in the infection of immune cells by wild-type MV [21] while Nectin-4 has been recently described as essential for host-to-host spread of MV [20, 22]. None of the tumor cell lines studied in our experiments exhibited CD150/SLAM expression (Figure 2(a)) and Nectin-4 was only found to be weakly expressed on HT29 cells (Figures 2(a) and 2(c)) as determined by flow cytometry.

### 3.4. *In Vivo* Oncolytic Activity of MV

**Figure 3:**
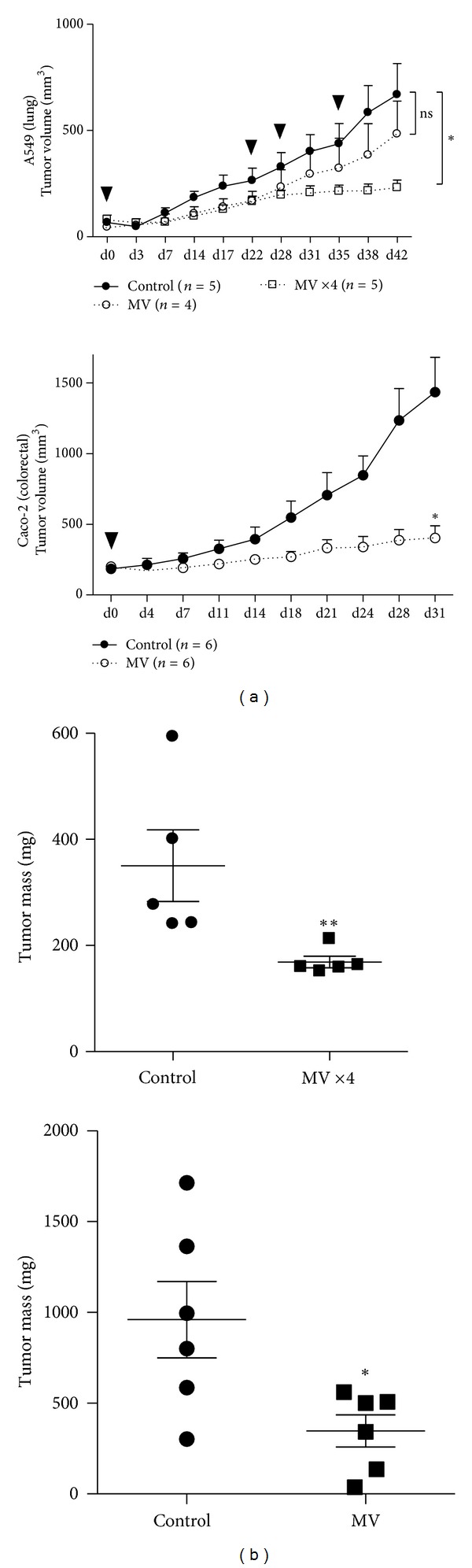
Measles virus exhibits oncolytic properties against human adenocarcinomas *in vivo. *(a) Nude mice were challenged subcutaneously with 1 million A549 (lung) or Caco-2 (colorectal) human adenocarcinoma cells. When tumors reached a volume of 100 mm^3^ for A549 or 150–200 mm^3^ for Caco-2, MV-eGFP or PBS was injected intratumorally (day 0, 1.5 × 10^7^ TCID_50_). Tumor volumes were measured twice weekly. Additional intratumoral MV injections were performed with A549 mice at days 22, 28, and 35 after initial injection as indicated by arrowheads. (b) When tumors reach 1–1.5 cm^3^ or after 31 (Caco-2 tumor) or 42 (A549 tumors) days, animals were sacrificed and tumors were harvested and weighed. (*P < 0.05; **P < 0.01).

To confirm our *in vitro* results regarding oncolytic properties of live-attenuated MV, we studied the ability of the virus to efficiently target and kill human lung and colorectal adenocarcinoma subcutaneous xenografts *in vivo* in nude mice (Figure 3). As our ultimate goal is to develop therapy against aggressive established tumors, we decided to treat large (100 to 200 mm^3^) A549 and Caco-2 tumors.

Human Caco-2 colorectal adenocarcinomas xenografts were treated by a single intratumoral injection of oncolytic MV (1.5 × 10^7^ TCID_50_). Whereas we previously observed slow transmission of the virus* in vitro* for this cell line (Figure 1), a single injection of the virus resulted in growth arrest of the tumor for up to 31 days (Figure 3(a)). Conversely, tumor growth was constant in mice treated with control saline buffer (PBS). After 31 days, significant differences were observed between control and MV-treated mice regarding either tumor volumes (1434 ± 246 mm^3^ versus 403 ± 86 mm^3^; *P* < 0.05; Figure 3(a)) or tumor weights (960 ± 211 mg versus 347 ± 89 mg; *P* < 0.05; Figure 3(b)).

Treatment of human A549 lung adenocarcinoma xenografts using identical experimental settings was not found to be effective. Indeed, even though MV induced a delayed tumor growth, we did not obtain any significant difference between PBS- (668 ± 147 mm^3^) and MV-treated (484 ± 154 mm^3^) mice (Figure 3(a)). This could be explained by the higher proliferation capacities of A549 cells as observed previously by time-lapse microscopy (Figure S2). Considering that steady proliferation of A549 cells could result in reduced viral particles/cells ratio into the tumor, we decided to carry out multiple injections of oncolytic MV by performing three extra intratumoral MV injections at days 22, 28, and 35 after the initial injection. This treatment schedule efficiently stopped tumor growth, as shown by the differences in tumor volumes between control animals (668 ± 147 mm^3^) and multiple-injections group (231 ± 36 mm^3^; *P* < 0.05) at day 42 (Figure 3(a)). At this point, tumors were weighed and tumor masses were found to be significantly different between control and MV-treated mice (350 ± 67 mg versus 169 ± 11 mg; *P* < 0.01; Figure 3(b)).

### 3.5. MV Infection Induces Caspase-3 Activation in Colorectal Tumor Cells

**Figure 4:**
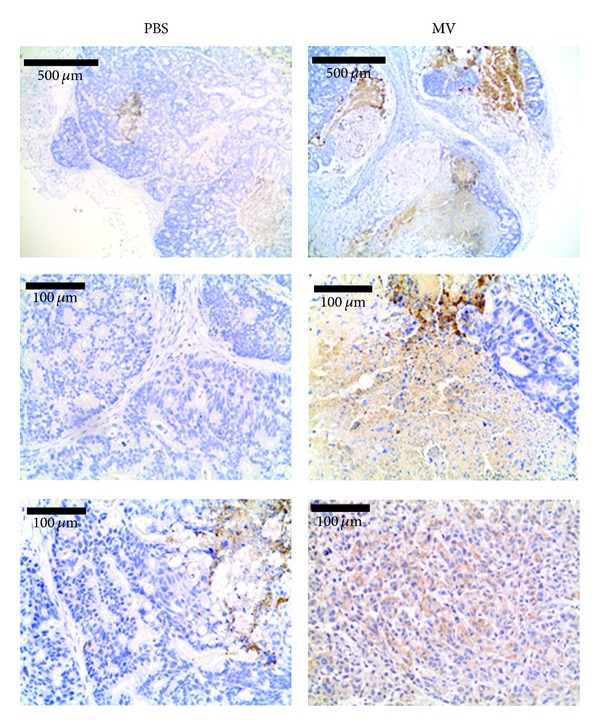
Caspase-3 activation in MV-treated colorectal adenocarcinomas. Representative sections of Caco-2 human tumor xenografts, 31 days after PBS or MV treatment. Tumors were analyzed for caspase-3 activation (brown staining) by immunohistochemistry as described in Section 2. Tumors were counterstained with hematoxylin to mark nuclei (blue staining).

Cell death triggered by MV infection has been previously described to be apoptosis [23]. Our results above are consistent with these observations as we found increased percentages of annexin-V^+^/PI^−^ lung and colorectal carcinoma cells following MV infection (Figure 1(c)). To investigate induction of apoptotic tumor cell death *in vivo*, we analyzed activation of caspase-3 in MV-treated and PBS-injected tumors by immunohistochemistry (Figure 4). Caspase-3 is involved in late events of apoptosis and thus can be activated by both extrinsic and intrinsic apoptotic pathways.

Caco-2 colorectal tumors grew in a specific way by forming round structures as shown by microscopy (Figures 1(b) and S1). In control mice, we observed minimal caspase-3 activation in the centers of these structures, which could be related to hypoxia or nutrient deprivation (Figure 4). In contrast, we observed strong activation of caspase-3 in MV-treated tumors, likely as a result of *in vivo* oncolytic activity of MV against Caco-2 colorectal adenocarcinoma cells. This strong and extended activation of caspase-3 throughout the tumor correlates with significant tumor growth arrest (Figure 3(a)). We did not observe any activation of caspase-3 in control and MV-infected A549 tumors (not shown).

## 4. Discussion

We report here that Schwarz live-attenuated vaccinal strain of measles virus (MV) exhibits both *in vitro* and *in vivo* antitumor activity against human lung and colorectal adenocarcinomas. MV was able to infect and efficiently induce cell death in different cell lines derived from these two types of cancers. Significantly, our *in vivo* experiments showed that MV is able to control the growth of large, established tumors derived from these human malignancies. These findings are of particular interest as lung and colorectal cancers are widespread aggressive diseases which are still in need of innovative antitumor treatments [24, 25]. Our work supports previous studies where oncolytic properties of MV vaccinal strains, derived from Edmonston lineage, have been demonstrated against a wide range of human cancers including lymphoma [26], glioblastoma multiforme [27], multiple myeloma [28], ovarian [29] and breast [30] carcinomas, prostate cancer [31], leukemia [32], and more recently melanoma [33].

MV-induced cell death has been characterized as apoptosis [23] and we regularly observed common features of apoptotic cell death, for example blebbing and early exposure of phosphatidylserine on the outer leaflet of plasma membrane. However, we also repeatedly observed atypical events that could possibly challenge the “apoptosis model”, at least when using live-attenuated MV against malignant cells. Indeed, we previously showed how mesothelioma cells infected with MV were able to induce spontaneous maturation of human monocyte-derived and plasmacytoid dendritic cells and, subsequently, the cross-presentation of tumor antigen to specific CD8^+^ T cells [6, 11]. Here, we observed that some of the infected cells undergoing cell death after syncytia formation showed rapid release of intracellular content into the culture medium, as shown by rapid leakage of cytoplasmic GFP. This could partially explain the immunogenicity of “apoptosis” that we harnessed in our previous work [6]. Recent advances in the characterization of tumor cell death pathways [34]—for instance description of necroptosis—could help to improve our comprehension of oncolytic MV-related cell death. Altered apoptotic pathways in tumor cells could also impact this process, as we observed divergent mechanisms in Caco-2 and A549 regarding syncytia formation *in vitro* and cleavage of caspase-3* in vivo*. However, the fact that we did not observe activated caspase 3 in A549 tumor in mice could be due to the rapid growth of this cell line compared to Caco-2. It is thus possible that rapidly dividing A549 cells outgrow activated caspase-3 apoptotic tumor cell and are thus more difficult to detect in histologic samples. Since more and more groups now focus on improving proapoptotic capacities of oncolytic viruses to circumvent resistance of tumor cells to cancer therapies [35], such information would be of great interest for clinical utility. Even if viruses are potent inducers of cell death and are often able to demonstrate cytotoxicity even in unfavorable conditions, it has been shown recently, for instance, that overexpression of antiapoptotic molecules from Bcl-2 protein family can impair MV-induced cell death in leukemia cells [32].

Better understanding of the features of MV-induced cell death will help to design appropriate clinical strategies for targeting aggressive human neoplasms. Antitumor effects of virotherapy have been shown to be enhanced by combination with other antitumor treatments in different models [36]. Combination with chemotherapy, the use of histone deacetylase inhibitors, for example, was found to improve the oncolytic properties of both vesicular stomatitis virus [37] and herpes virus [38]. Combinations with radiotherapy are also now widely described [39, 40]. Associations with immunotherapy, to combine direct oncolysis with an adaptive, antitumor immune response, is also an interesting alternative, with some groups demonstrating a critical role for the immune system in the therapeutic effects of oncolytic viruses [6, 41, 42]. Others have used engineered oncolytic viruses expressing proimmune molecules [5, 43]. Synergistic action of virotherapy and antitumor immune response *in vivo* has been extensively shown for oncolytic VSV [44] and reovirus [45, 46]. *In vitro* studies with MV tend to confirm this trend even though a suitable immunocompetent model to study interactions between the virus and the immune system is lacking [47]. For example, we (manuscript in preparation) and others [33] have observed the release of danger signals by MV-infected cells* in vitro*, although the *in vivo* consequences remain largely unknown.

Recent identification of Nectin-4 as a new receptor for measles virus has been a major step forward for a better understanding of MV biology [14, 20]. Nonetheless, CD46 membrane complement regulatory protein expression still remains the key determinant for cancer cell sensitivity (i) to primary infection by MV and subsequently (ii) to the efficient transmission of new viruses to neighboring cells by formation of multinucleated syncytia [48]. Overexpression of CD46 had been previously described in human lung and colorectal adenocarcinomas [10, 14] and we were able to confirm these observations in several cell lines derived from these two malignancies. Moreover, we showed that cell lines exhibiting high CD46 expression also displayed high infection rates by oncolytic MV. The virus was also able to induce substantial regressions of these human adenocarcinomas engrafted in nude mice. Surprisingly, one of our lung adenocarcinoma cell lines (ADK153) was found to be efficiently infected by MV while displaying a lower expression level of CD46 receptor (Figures 1 and 2). This unexpected observation supports preliminary experiments in melanoma where low expression of CD46 and the absence of SLAM and Nectin-4 receptors do not correlate with efficient infection by live-attenuated vaccinal MV (manuscript in preparation). This indicates that additional parameters have to be taken into account when tumor cells are screened for their susceptibility to oncolytic MV. Sensitivity to innate antiviral mechanisms—such as the ability to respond to Type-I interferon signaling—should be considered, even if one cannot exclude that, out of CD46, CD150/SLAM, and Nectin-4 receptors, other unidentified surface molecules could play a role in MV infection mechanisms. Nevertheless, the majority of the tumor cell lines we studied showed increase in sensitivity to oncolytic MV infection when overexpressing CD46, thereby showing that lung and colorectal cancers, which are highly resistant to conventional treatments, are suitable targets for MV cancer virotherapy.

## 5. Conclusions

Our experiments demonstrate that live-attenuated oncolytic MV is able to efficiently target human lung and colorectal carcinomas, thus highlighting novel options for the treatment of these aggressive malignancies. Further studies to better characterize cell death pathways activated by MV infection and parameters involved in MV susceptibility would again reinforce the interest in development of live-attenuated MV for cancer therapy.

## Supplementary Material

Figures S1 & S2: Analysis of adenocarcinoma cells infection by time-lapse microscopy. To analyze kinetics of infection by oncolytic measles virus, Caco-2 colorectal (Figure S1) and A549 lung (Figure S2) adenocarcinoma cells were infected with MV-EGFP (MOI = 1.0). Pictures were taken every 15 minutes for 72 hours using a time-lapse microscope (time is indicated as days:hours:minutes). Bright field and fluorescence pictures are merged.
